# Malaria causing post-infectious cerebellitis, a case report and literature review

**DOI:** 10.1016/j.amsu.2022.104462

**Published:** 2022-08-19

**Authors:** Mohamad Hijazi, ELMustafa Abdalla, Abdalla Fadul, Doaa Ahmed Eltayeb, Abdulrahman Al-Mashdali

**Affiliations:** Department of Internal Medicine, Hamad Medical Corporation, Doha, Qatar

**Keywords:** Post-infectious cerebellitis, Malaria, Parasitic infection

## Abstract

**Background:**

Malaria is a common parasitic infection with a wide range of clinical presentations. Thus, it should be suspected for any symptomatic patient with a recent travel history to a malaria-endemic area.

**Case presentation:**

In this case report, we will present a previously healthy 28-year-old patient who developed cerebellar signs that were attributed to infectious etiology later on proven to be a malaria-related complication that responded well to anti-malarial medications.

**Discussion:**

The classical presentation of malaria with attacks of fever is noticed only in around 60% of the patients. The development of immunity, the increasing resistance to anti-malarial drugs, and the indiscriminate use of anti-malarial drugs have led to malaria presenting unusual characteristics. Cerebellar ataxia, extrapyramidal signs, and various psychiatric manifestations have been described as early presentations of cerebral malaria or as a part of the post-malaria neurological syndrome. Other neurological syndromes like peripheral neuropathies, movement disorders, myelopathies, and stroke-like syndrome have also been described.

**Conclusion:**

Malaria should be included as a differential diagnosis in a patient presenting with cerebellar signs as its devastating if left untreated. However, it responds well to anti-malarial regimens if started early during the course.

## Highlights

• Malaria can cause a wide range of neurological manifestations.
• Plasmodium falciparum is the one associated with sever presentations.
• Post-infectious cerebellitis can be caused by malaria.
• Cerebellitis usually respond well to malaria treatment.

## 1 Introduction

Malaria is a common worldwide parasitic infection that has a wide range of clinical presentations. Because malaria has a wide variety of symptoms, it should be suspected and tested in any symptomatic patient with a recent travel to a malaria-endemic area. Although the cause is unclear, it can potentially interfere with our neurological function causing encephalitis, which can lead to death or neurological complications [1,2]. a significant portion of malarial neurological complications are caused by plasmodium falciparum, with cerebral malaria being the most dangerous. However, another rare neurological sequel has also been reported, including stroke and cerebellar ataxia [3,4]. Here we report a case of a young female who presented with post-infectious cerebellitis due to plasmodium falciparum malaria.

## 2 Case presentation

A 28 years old Sudanese female with a past medical history of asthma and on PRN inhalers and a recent malarial infection two months prior to this presentation was treated with chloroquine. She presented to the hospital with a one-week history of worsening epigastric abdominal pain exacerbated by food intake, non-radiating, associated with nausea and vomiting. She reported having mild headaches and dizziness for two days before the presentation. She was afebrile and vitally stable during her hospital stay. Her physical examination was unremarkable except for intension bilateral hands tremor, impaired finger to nose test, resting position jerky nystagmus, and, Subsequently, basic labs were utterly unremarkable, including complete blood count, renal function tests, liver function tests, and C-Reactive Protein. The neurology team labeled her as a case of post-infectious cerebellar syndrome. Subsequently, her viral screening, including EBV and CMV, was unremarkable. However, her Blood film showed ring forms and gametocytes of Plasmodium falciparum at 0.1%. Ultrasound abdomen showed spleen enlargement of 16 0.1 cm, but an MRI Head both with and without contrast was unremarkable. The infectious diseases team started her on artesunate for five days with subsequent significant symptomatic improvement. Upon discharge, she was asymptomatic and returned to her baseline health status.

## 3 Discussion

Malaria is a common parasitic illness caused by a protozoan from the genus ‘Plasmodium’ of which there are four human species: Plasmodium falciparum, vivax, ovale, and Plasmodium malaria. Malaria causes clinical sickness in around 300–500 million people worldwide, and over 1 million people die annually. In India, malaria incidence since 1982 is about 2 million cases per year, representing 40% of the whole number of cases outside Africa. Falciparum malaria proportion is between 35 and 43%, which affects both young and old individuals, but children are particularly at risk [5]. Several neurological presentations of malaria have been reported. Of all malarial parasites that infect humans, Plasmodium falciparum is most commonly associated with neurological complications. Cerebellar involvement also can happen, but it is an unusual complication of falciparum malaria [6]. Cerebellar involvement can be associated with complicated and uncomplicated malaria. The pathophysiology is likely due to Purkinje cells damage by hyperpyrexia [7]. Dominant cerebellar involvement could be part of cerebral malaria in which cerebellar signs resolve along with cerebral expressions [8]. A Post‐ Malaria Neurological Syndrome (PMNS) has been frequently reported. The most common signs and symptoms of PMNS included impaired consciousness, confusion, fever, seizure, weakness, headache, and ataxia. Most patients have an afebrile course before cerebellar symptoms and signs, which is always associated with Plasmodium falciparum infection. The exact mechanism of delayed cerebellar ataxia is unknown; however, there is some evidence to suggest the involvement of immunological factors in pathogenesis. [9] recent studies indicate cerebral malaria likely involves a complicated imbalance of pro-inflammatory cytokines. Also, there is evidence that cytoadherence of PRBCs in the brain microvasculature may be a secondary effect of the “cytokine storm” in intense falciparum infection [10]. Regarding investigations, in 11 cases in which an EEG was completed, it demonstrated mild to severe encephalopathy (diffuse background slowing). The range of reported Glasgow Coma scale was 6–14. MRI showed abnormalities in 4 out of 12 cases in which it was performed. Abnormalities increased signal uptake in various regions, including the periventricular areas, pons, thalamus, corona radiata, internal capsule, and cerebellum. For all patients in which the MRI had acute abnormalities, CT had also been performed and was unremarkable. Moreover, CT failed to reveal any acute abnormalities in any of the 12 cases in which it was conducted. CSF analysis was performed in every case, revealing a lymphocytic pleocytosis (>5 lymphocytes, range 10–76) in 18 cases (50%) and elevated protein in 24 cases (69%) [11]. This condition is self-limiting, mainly with a good prognosis requiring only *anti*-malarials medications and symptomatic treatment, with a few cases requiring steroids [12]. Thus, practicing clinicians in endemic malaria areas should include neurological complications of P. falciparum malaria as a differential diagnosis in all patients with cerebellar symptoms. Despite cases of neurological manifestations of P. falciparum malaria being reported, WHO has not included the various presentations of neurological complications of P. falciparum malaria other than cerebral malaria. This highlights the need to study all neurological manifestations of P. falciparum malaria properly. Its treatment protocol needs to be done and revised to help the Attending physician recognize and manage the illness and its complications at the earliest to improve the disease's outcome.

This work has been reported in line with the SCARE 2020 criteria [13].

## 4 Conclusion

Neurological manifestations are essential to the clinical features of complicated and uncomplicated malaria. Neurological involvement is commonly associated with falciparum malaria because its unique characteristics lead to microvascular involvement. Both the central and peripheral nervous system is likely to be involved. Neurological side effects of anti-malarial medications can be added to the spectrum of neurological manifestations. Rational use of anti-malarial drugs will go a long way to preventing devastating neurological sequelae and neurological complications of malaria. We are reporting this case to improve physicians' awareness of the rare cerebellar complications of malaria and to adhere to the recommendations of WHO for proper use of these newer and more potent anti-malarial drugs.

## Ethical approval

The case report was approved by Hamad Medical Corporation Medical Research Centre.

Reference number: **MRC-04**–**22**–**393**.

## Sources of funding

Open access funding was provided by Qatar National Library (QNL).

Role of sponsors: No role in our study.

## Author contribution

Mohamad Hijazi identified the case, reviewed the literature, and wrote the manuscript. ELMustafa Abdalla is the corresponding author who helped in manuscript writing, doing a review for literature.

Abdalla Fadul, Abdulrhman AL-Mashdali, and Doaa Ahmed Eltayeb, helped in identifying the case, reviewing the literature, and doing the final review and approval for the manuscript.

## Conflicts of interest

The authors have no competing of interest to declare.

## Registration of research studies

Not required.

## Guarantor

ELMustafa Abdalla.

## Consent

Written informed consent was obtained from the patient for publication of this case report and the accompanying image. A copy of the written consent is available for review by the Editor-in-Chief upon request.

## Consent for publication

Written informed consent was obtained from the patient for publication of this case report and the accompanying image. A copy of the written consent is available for review by the Editor-in-Chief upon request.

## Availability of data and materials

The datasets used and/or analyzed during the current study are available from the corresponding author on reasonable request.

## Provenance and peer review

Not commissioned, externally peer-reviewed.
